# The *N*-Glycosylation Processing Potential of the Mammalian Golgi Apparatus

**DOI:** 10.3389/fcell.2019.00157

**Published:** 2019-08-13

**Authors:** Peter Fisher, Jane Thomas-Oates, A. Jamie Wood, Daniel Ungar

**Affiliations:** ^1^Department of Biology, University of York, York, United Kingdom; ^2^Department of Chemistry and Centre of Excellence in Mass Spectrometry, University of York, York, United Kingdom; ^3^Department of Mathematics, University of York, York, United Kingdom

**Keywords:** computational modeling, Golgi apparatus, glycan biosynthesis, cisternal number, glycan heterogeneity

## Abstract

Heterogeneity is an inherent feature of the glycosylation process. Mammalian cells often produce a variety of glycan structures on separate molecules of the same protein, known as glycoforms. This heterogeneity is not random but is controlled by the organization of the glycosylation machinery in the Golgi cisternae. In this work, we use a computational model of the *N*-glycosylation process to probe how the organization of the glycosylation machinery into different cisternae drives *N*-glycan biosynthesis toward differing degrees of heterogeneity. Using this model, we demonstrate the *N*-glycosylation potential and limits of the mammalian Golgi apparatus, for example how the number of cisternae limits the goal of achieving near homogeneity for *N*-glycans. The production of specific glycoforms guided by this computational study could pave the way for “glycoform engineering,” which will find uses in the functional investigation of glycans, the modulation of glycan-mediated physiological functions, and in biotechnology.

## Introduction

**FIGURE 1:**
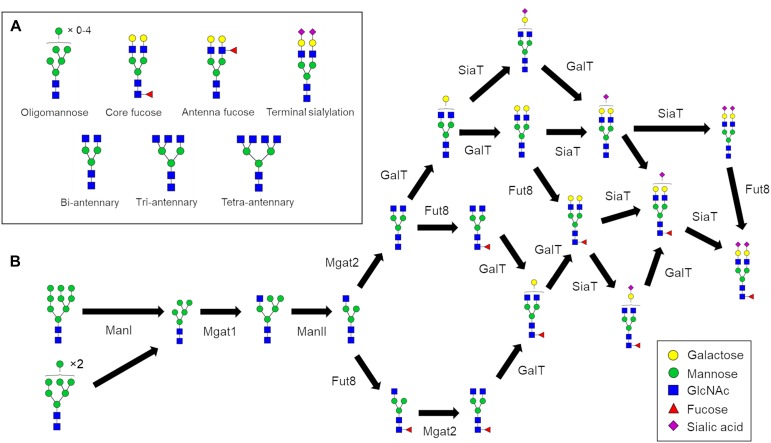
*N*-glycan processing pathways to generate a fully sialylated bi-antennary glycan. **(A)** Pictorial examples of structural features of complex *N*-glycans that are referred to in this work. **(B)** *N*-glycan biosynthesis pathway for Fuc_1_GlcNAc_4_Man_3_Gal_2_NeuAc_2_ starting with the two oligomannose glycans that enter the Golgi apparatus. The biosynthesis of complex *N-*glycans is characterized by increasing numbers of reaction paths that ultimately converge on structural nodes often due to the addition of terminating sialic acids.

*N*-glycosylation is a process initiated in the endoplasmic reticulum (ER) but specific structural elements such as core fucosylation and branching (Figure 1A) are introduced later in the secretory pathway in the Golgi apparatus. *N*-glycans are modified by a series of sequentially acting glycosidases and glycosyltransferases (Figure 1B) that modify glycans in the Golgi apparatus and consequently dictate and ultimately determine the glycan profile of the whole cell. However, the structural modification of *N*-linked glycans is a complex process that results in numerous different glycan structures. In the absence of a “glycan template,” protein glycosylation is inherently heterogenous with a number of factors contributing to the final glycan structure. These variables include the protein structure (Hang et al., 2015; Suga et al., 2018), secretory protein load (Jimenez-Mallebrera et al., 2009), Golgi transport mechanism (Hossler et al., 2007), enzyme protein levels, availability of monosaccharide-nucleotides, and the organization of glycosylation enzymes within the Golgi apparatus (Oka et al., 2004; Zolov and Lupashin, 2005; Fisher and Ungar, 2016). Typically, *N*-glycan biosynthesis can be characterized as a series of divergent pathways that converge into structural nodes (Figure 1B). Following the initial trimming of mannoses by ManI, the number of possible *N*-glycan structures generated in the Golgi apparatus increases exponentially with each additional monosaccharide until the capping of antennae with sialic acid (Spahn et al., 2016).

*N*-glycans can play important roles in dictating the properties of glycoproteins. From a biologic’s standpoint, controlling the glycans residing on therapeutic antibodies can tune their pharmaceutical properties. For example, glycans lacking core fucose (Figure 1A) can increase antibody-dependent cellular cytotoxicity (ADCC) (Shinkawa et al., 2003; Yamane-Ohnuki et al., 2004). Overall it appears that fucosylation and galactosylation are the dominant features of *N*-glycans that influence Fc-receptor binding (Dekkers et al., 2017), which is linked to ADCC. Furthermore, the presence of sialylated complex type glycans increases the *in vivo* half-life of therapeutic antibodies compared to oligomannose type glycans (Kanda et al., 2007) further illustrating the attenuating role of *N*-glycans in IgG properties. Due to the heterogenous nature of glycan biosynthesis the production of homogenous biologics with respect to glycosylation is currently not feasible, giving rise to batch-to-batch variation, and heterogeneity within batches. Glyco-engineering of biologics, for example through the deletion of Mgat1 to eliminate complex and hybrid glycans from CHO cells, reduces glycan heterogeneity, and improves immunological properties (Xu et al., 2016). However, eliminating complex glycans also removes any ability to fine-tune the properties of a therapeutic through presence/absence of other *N*-glycan features such as those shown in Figure 1A. Therefore, devising novel strategies to reduce glycan heterogeneity and/or enrich the relative abundance of a desired glycan is of great value to the pharmaceutical industry.

Glycosylation enzyme levels play a pivotal role in glycan biosynthesis; however, the outcome of enzyme depletion or overexpression can be unexpected. Knockdown of the galactosyltransferase GalT4 alongside overexpression of the branching enzymes Mgat4 and/or 5 in CHO cells substantially increased the abundance of tri- and tetra-antennary *N*-glycans. Interestingly though, it was the reduction of GalT4 that primarily accounted for the increase in branching (McDonald et al., 2014). Two additional important factors in determining the *N*-glycans produced by mammalian cells are the distribution of the glycosylation enzymes within the Golgi apparatus (Fisher and Ungar, 2016), and the architecture of the Golgi apparatus itself. For example, disruptions to the conserved oligomeric Golgi (COG) complex, which is involved in tethering and thereby targeting intra-Golgi retrograde vesicles, can lead to glycosylation defects in model cell lines (Shestakova et al., 2006; Bailey Blackburn et al., 2016) and to congenital disorders of glycosylation (reviewed in Wu et al., 2004; Steet and Kornfeld, 2006; Hennet and Cabalzar, 2015). Furthermore, the depletion of the Golgi reassembly stacking proteins (GRASPs) of 55 and 65 kDa results in an acceleration of protein transport, Golgi fragmentation, and impaired glycosylation (Xiang et al., 2013; Bekier et al., 2017). Fragmentation of the Golgi apparatus has also been linked to numerous neurodegenerative disorders (reviewed in Joshi et al., 2015). Alterations to the architecture and the organization of the Golgi apparatus provide a potential route to controlling glycosylation alongside the protein levels of the glycosylation machinery themselves. Other factors such as the availability of the monosaccharide-nucleotide donors are important and worthy of further investigation but are not considered in this work.

In this work we build on previous studies of glycosylation in WT HEK293T cells (Bailey Blackburn et al., 2016) and a stochastic model of *N*-glycosylation we recently developed and validated (Fisher et al., 2019). We used this model of glycosylation to demonstrate *in silico* the effects of cisternal number on glycan heterogeneity and complexity. This led us to test computationally what limitations the existing Golgi apparatus architecture places on the degree of glycan homogeneity achievable in mammalian cells. Finally, we used our model to predict strategies to increase the relative abundance of targeted glycan structures.

## Results and Discussion

It is not the aim of this work to describe in great detail the modeling methodology that has been used (for a more detailed description on the development and validation of the modeling framework used in this work please see Fisher et al., 2019), however, a brief summary of the modeling methodology will give context and a greater understanding of the results of this work. Glycosylation reactions are simulated using a stochastic simulation algorithm (SSA). The SSA incorporates the inherent noise that is present in biological systems and that becomes increasingly relevant when the number of reactants (and enzymes) are small, as is the case for glycosylation in the Golgi. In contrast to models of glycosylation based on ordinary differential equations (ODEs) (Hossler et al., 2007; Krambeck et al., 2009, 2017; Goey et al., 2018), several factors can be included in each parameter, reducing the need for excessive parameterization in our model. As such, we define parameters for each enzyme by using the term “effective enzymatic rate” to encompass the enzyme’s protein level, availability of its nucleotide-monosaccharide substrates and the chemical enzymatic rate. In this work we do not wish to consider changes to intrinsic enzymatic properties such as the inherent chemical enzymatic rate constants, therefore any predicted alterations to the effective enzymatic rate parameter are assumed to be equivalent to alterations in the protein level of the respective enzyme.

The effective enzymatic rates, the transit time in the Golgi apparatus, and the composition of glycans entering the Golgi were the parameters that were altered to fit the computed to a target glycan profile. This fitting was done using an approximate Bayesian computation (ABC) algorithm (Marjoram et al., 2003), which samples from parameter distributions to feed the SSA and assess the goodness of fit with a target profile. The shift of the parameter distributions during fitting tells us how the organization (e.g., levels and localizations) of enzymes changes from the starting condition to reach the target glycan profile. We utilize the optimized parameters determined previously for the WT HEK293T cell glycan profile as initial values (Fisher et al., 2019), therefore predicted changes in effective enzymatic rates are relative to the organization of the glycosylation machinery in WT HEK293T cells.

### Glycan Heterogeneity and the Golgi Apparatus

**FIGURE 2:**
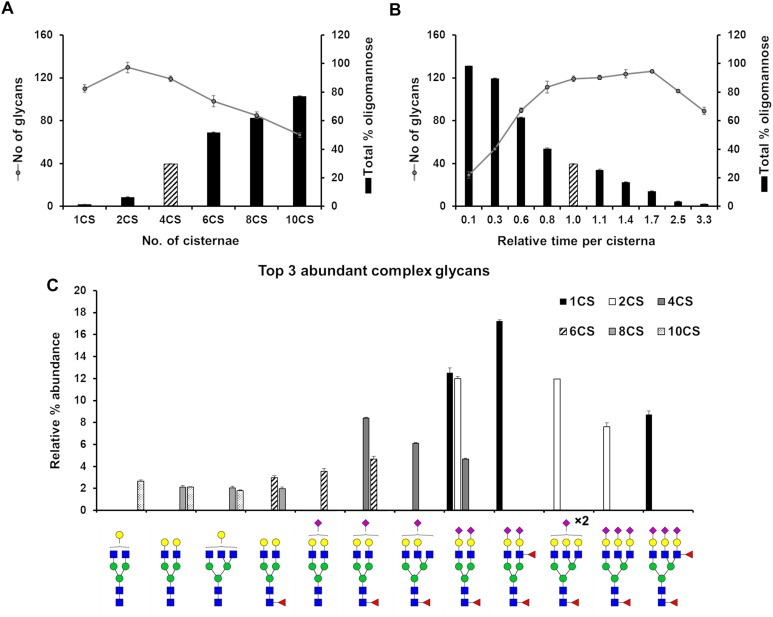
Effect of cisternal number and transit time on glycan heterogeneity. **(A)** Total number of glycans (circles) and proportion of oligomannose glycans (bars) produced *in silico* when starting with parameters fitted for the WT HEK293T cell glycan profile altering the number of cisternal elements (CS) in the system from the four (striped bar) used in the WT HEK293T model. The distribution of enzymes across the model Golgi apparatus, the total effective rates for each enzyme and the total time spent in the Golgi apparatus was kept the same for each simulation. **(B)** As in “A” but varying the total time spent in the Golgi with four cisternae. Striped bar indicates transit time for the WT HEK293T cell model; the times shown for all other simulations are relative to this value. **(C)** Relative proportions of the three most abundant complex glycans predicted by the model Golgi apparatus when the number of cisternal elements are altered. For all simulations error bars show standard deviation for *n* = 3.

We set out to evaluate how the level of glycan heterogeneity is affected by different variables in our model of *N*-glycosylation. An important variable is the number of cisternal elements, which is known to vary between species, cell types (Mironov et al., 2017), and in pathologies (Joshi et al., 2014). In our earlier work four cisternae were found to be required to optimally model HEK293T cell *N*-glycans (Fisher et al., 2019). While this number does not necessarily reflect the actual number of cisternae in this cell type, deviations from this number during modeling reflect potential changes in cisternal number. In order to investigate the effect of architecture on the glycan processing potential of the Golgi apparatus, the number of cisternal elements in our stochastic model of the Golgi was varied from the four required for HEK293T cells in previous work (Fisher et al., 2019). The total effective rate for each enzyme was kept constant, as was the total time spent in the Golgi apparatus. As a measure of glycan heterogeneity in these simulations, the number of different glycan structures produced in the simulation was recorded (Figure 2A). The trend is of decreasing heterogeneity as the number of cisternal elements is increased from two to ten. In addition to the observed decrease in heterogeneity correlating with increasing cisternal elements, the relative abundance of oligomannose glycans also increases as the trimming of mannose residues becomes less efficient possibly due to the reduced time per cisterna. The reduction in glycan maturity observed in GRASP55/65 double knockout (KO) cells may therefore be a consequence of the fragmented Golgi apparatus (Bekier et al., 2017). Interestingly, by changing the number of cisternal elements the most abundant complex glycans simulated by our model also differed (Figure 2C). For example, and in support of the hypothesis that the organization of a fragmented Golgi apparatus may explain the reduction in glycan maturity in GRASP55/65 KO cells, simulations with more than four cisternal elements generated immature complex glycans in comparison to simulations involving one and two cisternal elements, which generated higher relative abundances of more elaborate glycans containing more antennae, and a higher proportion of sialic acids (Figure 2C). This is possibly a knock-on effect of the reduced efficiency of oligomannose processing as mentioned above, and could possibly be explained with the shorter processing time in each individual cisterna.

In the previous simulation, the total time taken to traverse the Golgi apparatus was kept constant while the number of cisternal elements was altered. Next, we were interested in what effect varying the transit time per cisterna had on glycan heterogeneity and complexity while keeping the number of cisternae at four. Indeed, altering the time taken to traverse the Golgi apparatus had a different effect than varying the number of cisternal elements (Figure 2B). The number of different glycan structures predicted when the time was reduced to 10% of the WT time decreased dramatically (Figure 2B). Following a gradual increase and plateauing of glycan heterogeneity with increasing time in the Golgi apparatus, the number of glycan structures begins to decrease again at 250% of WT time. This is due to glycan pathways converging on structural nodes with terminating sialic acids. For example, the abundance of the fully sialylated bi-antennary glycan, Fuc_1_GlcNAc_4_Man_3_Gal_2_NeuAc_2_, increases from 4.5% to close to 10% of the total when the transit time is increased 3.3-fold of that of WT. In contrast, this same glycan is absent from the simulated profile when the transit time is reduced 10-fold compared to WT.

Interestingly, GRASP55/65 depletion accelerates protein trafficking through the Golgi apparatus (Xiang et al., 2013; Bekier et al., 2017), an effect that our model would predict to decrease the relative abundance of complex *N*-glycans (Figure 2B). Indeed, published experimental evidence shows less processing for both cell surface and intracellular glycans in these cells (Bekier et al., 2017). In contrast, there was a minor kinetic delay for the delivery of VSV-G to the plasma membrane in Cog3 knockdown HeLa cells (Zolov and Lupashin, 2005), indicating a reduced rate of anterograde transport. This is unlikely to be the direct cause of the glycosylation defect though, as Cog3 depletion results in relocation and partial degradation of Golgi glycosylation enzymes (Shestakova et al., 2006), as opposed to GRASP55/65 depletion that had no effect on the localization and levels of the glycosylation enzymes studied (Xiang et al., 2013). Furthermore, our model suggests slower transport through the Golgi apparatus would reduce the oligomannose content, a feature that is not observed in COG subunit depleted cell lines (Bailey Blackburn et al., 2016).

### Maximizing the Relative Abundance of Target Glycans

The production of single glycoforms is an important goal in the pharmaceutical industry. We therefore used our modeling framework to assess the glycan processing potential of the Golgi apparatus. In this instance, we tested the potential of the Golgi apparatus to produce a single glycoform, within reasonable biological boundaries. Three complex glycans with partial or complete sialylation and two or three antennae (Table 1) were chosen to test this property of the Golgi apparatus. The three complex glycans are referred to as bi-Sia_1_, bi-Sia_2_, and tri-Sia_1_ when they are described as the target for glycan engineering. When the same structures appear in the simulation as by-products though, they are referred to in conventional glycan notation. Starting with the fitted parameters for the effective enzymatic rates for WT HEK293T cells, we used ABC fitting to maximize the relative abundance of each target glycan. For this purpose, a hypothetical glycan profile was fitted in which 100% of the complex glycan was set as the desired *N*-glycan. The parameters that were fitted for these experiments were: effective enzymatic rates, transit time per cisterna and composition of glycans entering the Golgi. In addition, we investigated the maximization of target glycans in two scenarios: One, in which the distribution of enzymes between Golgi cisternae can be varied (variable localization); and another one, in which the distribution of enzymes in the Golgi apparatus is fixed to that observed in WT cells (fixed localization).

**TABLE 1:** Target glycans.

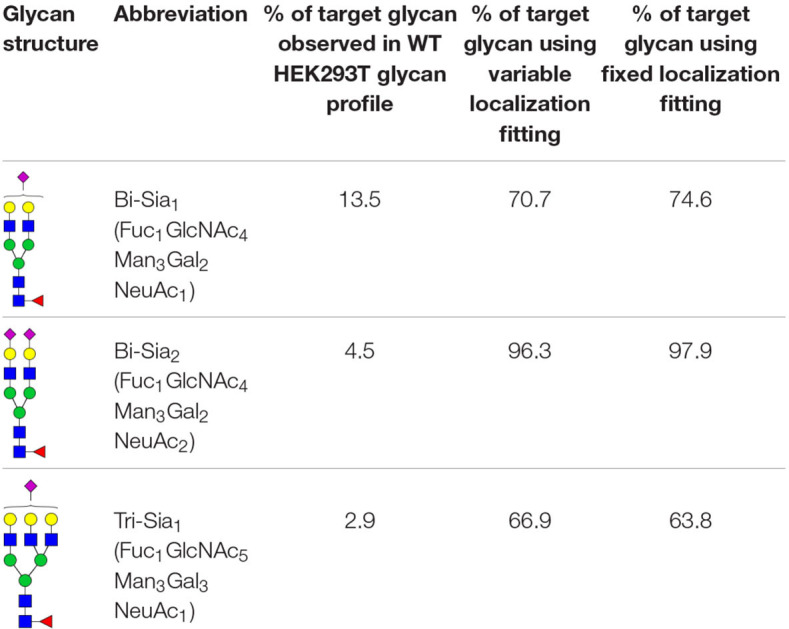

Table 1 shows the percentage of the target glycan normalized to the total amount of complex glycan observed experimentally in WT HEK293T cell samples (Bailey Blackburn et al., 2016). For all of the target glycans the proportion could be increased above that found in WT HEK293T cells (Table 1). Bi-Sia_2_ could be produced by the simulation at the highest relative abundance due to the terminating sialic acids on both antennae, which are endpoint structures. In contrast, homogeneous production of partially sialylated glycans is predicted to be much more difficult if not impossible to achieve according to our stochastic model. When comparing the fits to the bi-Sia_1_ and tri-Sia_1_ targets we can also say that adding a third antenna makes the drive to homogeneity again more difficult, implying that the inclusion of tri- and/or tetra-antennary glycans in products will inadvertently increase heterogeneity, as was observed in glycan-engineered plant expression systems (Nagels et al., 2012).

**FIGURE 3:**
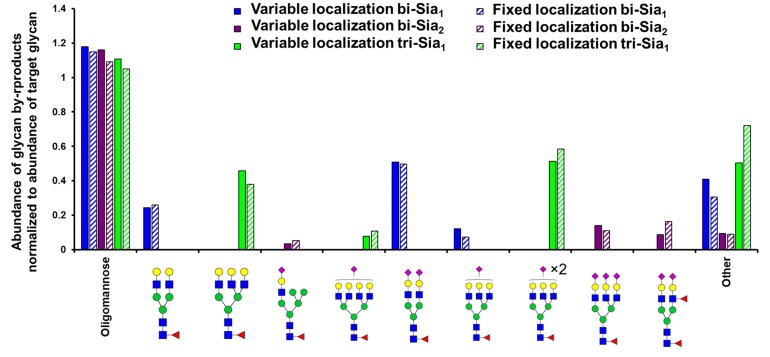
Glycan by-products of maximizing target complex glycans. Relative abundance of the oligomannose pool and the three most abundant complex glycans that are predicted as by-products when maximising the abundance of the indicated target glycans with the indicated type (fixed or variable enzyme localization) of fitting strategy.

As expected, the most abundant glycan by-products predicted when maximizing the production of each target glycan were quite different (Figure 3). When maximizing the abundance of bi-Sia_1_, the major by-products were the un-sialylated bi-antennary glycan Fuc_1_GlcNAc_4_Man_3_Gal_2_ and the fully sialylated Fuc_1_GlcNAc_4_Man_3_Gal_2_NeuAc_2_. This result suggests a small window in which the glycoprotein must exit the Golgi apparatus following the initial sialylation but prior to the second sialylation reaction to achieve a bi-Sia_1_ glycan structure. The result is similar when enriching tri-Sia_1_, as the dominant predicted by-products are the over-sialylated tri-antennary glycan, Fuc_1_GlcNAc_5_Man_3_Gal_3_NeuAc_2_, and the under sialylated tri-antennary glycan, Fuc_1_GlcNAc_5_Man_3_Gal_3_ (Figure 3). The major by-products for bi-Sia_2_ were the fully sialylated tri-antennary glycan, Fuc_1_GlcNAc_5_Man_3_Gal_3_NeuAc_3_ suggesting that the modeled sialyation activity is not limiting the production of bi-Sia_2_, rather the presence of branching enzymes is diverting flux away from bi-Sia_2_. These by-products could of course be eliminated by modeling a cell line in which the branching enzymes are completely removed, but the ABC algorithm has difficulties pushing enzyme levels to complete elimination i.e., zero, as it will always sample from a non-zero distribution.

**FIGURE 4:**
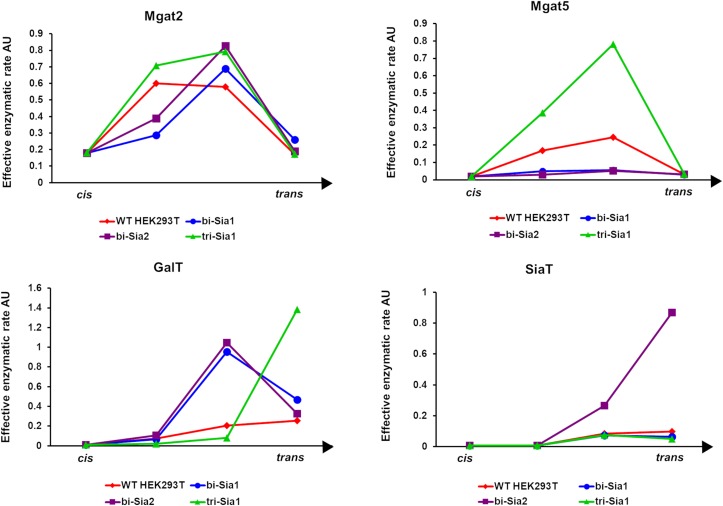
Optimized Golgi distribution of enzymes to maximize the relative abundance of target glycans. Distributions of the effective enzymatic rates for the indicated enzymes throughout the Golgi apparatus following fitting with variable enzyme localization to maximize the abundance of the indicated target glycans. The predicted distribution in WT HEK293T cells (Fisher et al., 2019) is shown for comparison.

**FIGURE 5:**
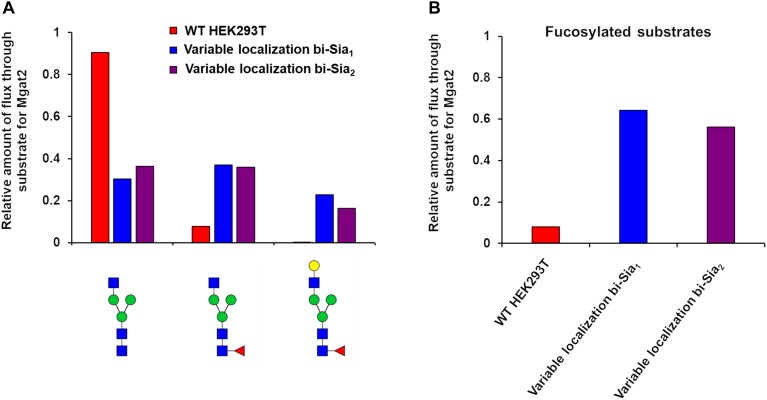
Flux analysis of Mgat2 substrates in WT, bi-Sia_1_, and bi-Sia_2_ simulations. **(A)** Proportion of biosynthetic flux carried by Mgat2 from its three dominant substrates in WT HEK293T cells (red) and the simulated profiles maximizing bi-Sia_1_ (blue) and bi-Sia_2_ (purple) fitted with variable enzyme localization. **(B)** The proportion of substrates for Mgat2 that are fucosylated for the simulations described in “**A**.”

Having started with the WT HEK293T cell parameters (Fisher et al., 2019), relative changes in enzyme distributions between Golgi cisternae that maximize the target glycans *in silico* can be predicted when using localization dependent fitting (Figure 4). For all target glycans an increase in the Mgat1 effective enzymatic rate within the second cisterna is required, and a similar change is seen for Fut8 (Figure 4). Both of these enzymes act early in the generation of complex *N*-glycans (Calderon et al., 2016; Fisher et al., 2019). The increases in their levels in an early cisterna presumably ensures that glycan processing can proceed more completely toward the desired products without the accumulation of partially processed intermediates. For the two bi-antennary glycan targets Mgat2 was required to be redistributed toward the *trans* side of the Golgi apparatus relative to the simulated WT distribution (Figure 4). Analysis of the flux map revealed that the shift in Mgat2 toward the *trans* side of the Golgi apparatus changed the dominant substrates for Mgat2 (Figure 5A). For the model of WT *N*-glycosylation only ∼8% of the Mgat2 substrates were fucosylated. In contrast, more than 60% of the Mgat2 substrates were fucosylated in the model primed to maximize the abundance of bi-Sia_1_, and a similar shift in substrate preference is seen for bi-Sia_2_ (Figure 5B). Unsurprisingly, the effective enzymatic rate of the branching enzyme Mgat5 was predicted to be increased when trying to maximize the abundance of tri-Sia_1_; however, the distribution of Mgat5 was also required to shift more to the *trans* side of the Golgi apparatus (Figure 4). This shift in Mgat5 coincided with an equivalent shift of GalT into the *trans*-Golgi, a characteristic that aims to spatially separate the two enzymes within the Golgi apparatus (Figure 4). This is in line with competition between galactosylation and glycan branching, a feature that was suggested to explain the demonstrated role of GalT in determining and controlling antenna number (McDonald et al., 2014; Fisher et al., 2019).

**FIGURE 6:**
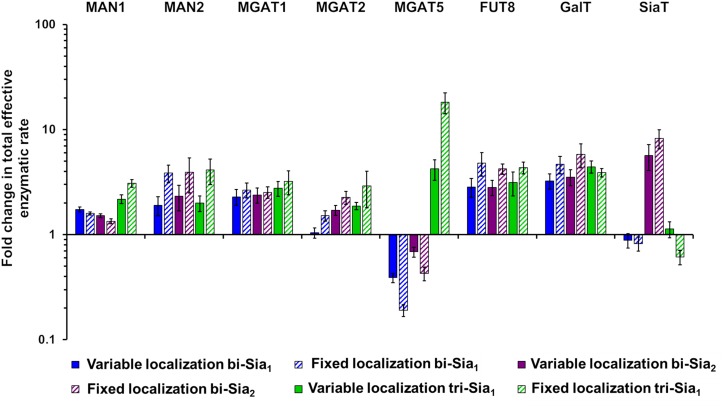
Total enzymatic rate changes to maximize relative abundance of target glycans. Percentage changes in total effective enzymatic rates following fitting to maximize the abundance of bi-Sia_1_ (blue), bi-Sia_2_ (red), and tri-Sia_1_ (green) fitted with variable enzyme localization (solid) and fixed enzyme localization (striped) relative to the parameters predicted for the WT HEK293T cell profile. Total effective enzymatic rates obtained from the different fits were adjusted to ensure that the transit time in each case matched that of the WT HEK293T simulation.

If we assume that the intrinsic rates of the glycosylation enzymes are not altered, predicted changes to the effective rates that are made by our model can be rationalized as overexpression or knock down of the relevant protein levels. Such an approach to reducing glycan heterogeneity and enriching the target glycan has indeed been previously applied experimentally for the simple case of core fucosylation (Yamane-Ohnuki et al., 2004). Figure 6 shows the predicted changes in total effective rate for each enzyme necessary to maximize the relevant target glycan. The differences in total effective enzymatic rates between the fits using variable and fixed enzyme localizations suggest that the distribution of enzymes is an important factor to be considered when designing genetic manipulations of the glycosylation machinery. When the localization of enzymes is fixed, predicted alterations to the total enzymatic rates are much larger than those required in the variable localization scenario (Figure 6). For example, the increase required for Mgat5 is more than threefold higher for the fixed localization fitting of tri-Sia_1_ compared with the variable localization fitting of the same glycan. While simply altering the levels of enzymes could conceptually be much simpler through knock-down or overexpression, their distribution may also be changed through engineering of the intra-Golgi vesicular sorting pathway. For example, manipulation of the interactions of the COG vesicle tethering complex (Miller et al., 2013; Willett et al., 2013), or adjustments in the cytosolic and transmembrane domains of particular glycosylation enzymes could both be used to alter enzyme locations (Ferrara et al., 2006; Becker et al., 2018).

### A Computationally Informed Strategy for Producing Tetra-Antennary Glycans

**FIGURE 7:**
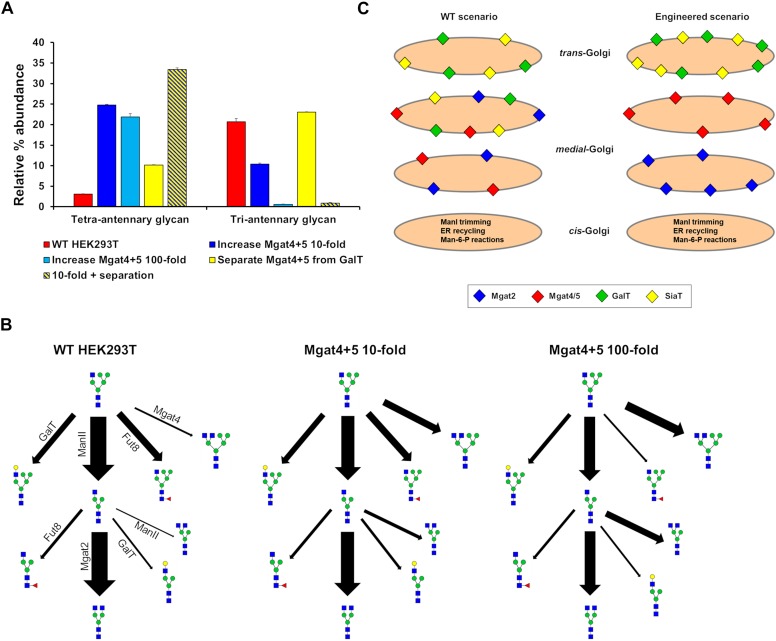
Hypothetical engineering of the Golgi apparatus to increase the abundance of tetra-antennary glycans. **(A)** The relative abundance of all tetra- and tri-antennary glycans produced *in silico* using effective enzymatic rates for: WT HEK293T (red), WT with the total effective enzymatic rates of Mgat4/5 increased 10-fold (dark blue), WT with the total effective enzymatic rates of Mgat4/5 increased 100-fold (light blue), WT with the distribution of enzymes in the model Golgi apparatus set to separate Mgat4/5 from GalT (yellow), and WT with the total effective enzymatic rates of Mgat4/5 increased 10-fold and the localization of enzymes in the model Golgi apparatus set to separate Mgat4/5 from GalT (yellow and dark blue striped). **(B)** Flux diagrams for selected *N*-glycosylation reactions for WT HEK293T, WT with the total effective enzymatic rates of Mgat4/5 increased 10-fold and WT with the total effective enzymatic rates of Mgat4/5 increased 100-fold. **(C)** Schematic showing the levels and relative localizations of key enzymes for WT HEK293T cells and the computationally engineered scenario predicted to maximize the production of tetra-antennary glycans.

Our ABC fitting strategy was unable to generate parameter values for the effective enzymatic rates that would simulate the production of significant amounts of tetra-antennary glycans. Initial parameter values for this fitting were those of the WT HEK293T cell line and therefore the huge shifts required in the effective rate of Mgat4 and 5 are almost never accepted within the fitting as they are considered highly unlikely relative to the WT values. However, it was possible to devise strategies to increase the output of tetra-antennary glycans from our model (Figure 7A). Manually elevating the effective rates of Mgat4/5 10-fold, while keeping the distribution of the enzymes the same as in WT HEK293T cells, increased the relative abundance of tetra-antennary glycans about sevenfold, to roughly 25% (Figure 7A). However, increasing the effective rates of Mgat4/5 further (100-fold) did not increase the relative abundance of tetra-antennary glycans above that of the 10-fold increased rates, but did increase the abundance of bi-antennary glycans (not shown). In order to investigate why further increasing the effective enzymatic rates of Mgat4/5 did not result in a concomitant increase in tetra-antennary glycans we examined the flux diagrams for the three conditions (Figure 7B). Flux analysis revealed that as the effective enzymatic rate of Mgat4 increases it begins to outcompete ManII and Mgat2 in the earlier cisternae resulting in a reduction in flux toward GlcNAc_4_Man_3_, which is an important intermediate on the path of tetra-antennary glycan biosynthesis (Figure 7B).

The enzymatic competition between ManII/Mgat2 and Mgat4 is predicted to limit the production of tetra-antennary glycans alongside the known effect of GalT on controlling the degree of *N*-glycan branching (Fisher et al., 2019). Elimination of GalT was suggested as an approach to producing a larger proportion of tetra-antennary glycans (McDonald et al., 2014). Spatially separating Mgat4/5 from GalT in the Golgi apparatus and therefore minimizing competition between the enzymes (Figure 7C) could achieve a similar effect for increasing the relative abundance of tetra-antennary glycans (Figure 7A). Indeed, the spatial separation of Mgat5 and GalT that is enforced by the model while enriching tri-Sia_1_ (Figure 4) demonstrates that cisternal separation allows GalT to evade dominating Mgat5. When applied to the task of increasing the proportion of tetra-antennary glycans the effect was not as large as increasing the effective rates of Mgat4/5, however, the two strategies were synergistic (Figure 7A). It is important to note that separation of the competing enzymes increased the relative abundance of tetra-antennary glycans but these were often not sialylated. Therefore, in this engineered theoretical scenario an additional cisterna to accommodate sialylation reactions may also be required depending on the target glycan.

## Conclusion

In this work our computational model of mammalian *N*-glycosylation has been used to probe the glycan processing potential of the Golgi apparatus. While this study was based on computation only, our model’s predictions generate some testable hypothesis, which will be interesting to follow up on using laboratory based experiments. Two aspects of Golgi biology, which are commonly affected in pathologies such as CDGs and neurological disorders, the architecture of the Golgi apparatus and transit time through the Golgi apparatus have been shown to be key determinants of the cellular glycan profile. Golgi architecture and transit time should be considered as important factors in glycosylation disorders that likely have an additive effect in disrupting the protein levels of the glycosylation enzymes.

The goal of generating homogeneous glycoforms of biologics from a process that is inherently heterogeneous is a difficult one. Our model suggests several strategies that we predict will enrich particular target glycan structures; however, our model could not achieve complete homogeneity of glycoforms, suggesting that full uniformity may not be achievable. In the cases of bi-Sia_1_ and tri-Sia_1_ a higher percentage of the target glycan could be achieved if the substrate specificities of the enzymes were treated as a variable and not fixed. This suggests that when partial sialylation (or galactosylation) is required, protein engineering to adjust the substrate specificities of enzymes is a necessary strategy. A combination of engineering glycosylation enzymes’ specificities, controlling effective enzymatic rates and organizing the Golgi apparatus may all be required to attain higher glycan homogeneity, but ultimately the number of cisternae in a mammalian Golgi will likely be a key limiting factor for producing large complex glycans close to homogeneity.

## Materials and Methods

### Modeling Framework

The fitted and validated model of HEK293T cells (Fisher et al., 2019) has been used extensively in this work. This model was presented in detail in Fisher et al. (2019); it is generated using a SSA based on the Gillespie algorithm (Doob, 1945; Gillespie, 1976) that was used to implicitly simulate enzyme competition in the Golgi apparatus. Glycans entered the *in silico* Golgi apparatus one at a time, hence our model does not account for substrate competition or the potential effects of protein load in the secretory pathway. The processing of 10,000 glycans was simulated in order to generate a simulated glycan profile from which the abundance of individual or structural classes of glycans can be obtained. This profile is compared to a real data set utilizing a Bayesian fitting methodology with priors based upon biological knowledge. In the case of this study the priors were based on the parameters obtained by fitting the WT HEK293T glycan profile.

### Transit Time and Cisternal Element Number

The enzymatic parameters as well as the proportions of Man_9_GlcNAc_2_, Man_8_GlcNAc_2_, and GlcMan_9_GlcNAc_2_ obtained for the fitted WT HEK293T cell line (Fisher et al., 2019) were used. The relative time taken for a glycan to transit through each cisternal element was varied and the glycosylation reactions simulated. The number of simulated glycans and the relative abundance of oligomannose glycans was calculated from the average of three simulations for each transit time.

For investigating the effect of cisternal element number on the *N*-glycosylation process the total effective enzymatic rates for each enzyme were kept constant in addition to the total transit time and proportions of Man_9_GlcNAc_2_, Man_8_GlcNAc_2_, and GlcMan_9_GlcNAc_2_. Extra cisternal elements were added by calculating the average effective enzymatic rate between two adjacent cisternae and then rescaling the effective enzymatic rates to equal the total for the fitted WT HEK293T cells. By using this strategy, we ensure any alterations to the simulated glycan profile are not the result of changes in enzyme levels. The number of simulated glycans and the relative abundance of oligomannose glycans was calculated from the average of three simulations for each cisternal number.

### Maximizing Target Glycans

To predict alterations to enzyme levels and localization that can maximize a given target glycan we used the ABC fitting methodology to fit a simulated glycan profile to a hypothetical observed glycan profile. The hypothetical glycan profile was constructed by making the relative abundance of the target glycan to 100% of the combined complex and hybrid glycan abundances. The proportion of oligomannose glycans was kept at the level observed in WT HEK293T cells in all target profiles. ABC fitting methodology was then used to predict alterations in the organization of the Golgi machinery to generate the hypothetical glycan profile. The model was not penalized for what type of by-products were produced, as such it is reasonable to assume that the predicted by-products would be observed in a true glycan profile of an engineered cell line.

### Maximising Tetra-Antennary Glycans

WT HEK293T fitted parameters were used as a starting point. The effective enzymatic rates of Mgat4 and Mgat5 were increased 10- or 100-fold and the glycan profile simulated. For the spatial separation of the key enzymes involved in determining tetra-antennary glycans, the effective enzymatic rates were condensed into specific cisternae as shown in Table 2. The relative abundance of all tetra-antennary glycans was calculated from the average of three simulations for each different scenario.

**TABLE 2:** Distribution of Mgat2, Mgat4, Mgat5, and GalT in an engineered Golgi apparatus to maximize tetra-antennary glycans.

**Enzyme**	**% effective enzymatic rate in 1st cisterna**	**% effective enzymatic rate in 2nd cisterna**	**% effective enzymatic rate in 3rd cisterna**	**% effective enzymatic rate in 4th cisterna**
Mgat2	0	100	0	0
Mgat4	0	0	100	0
Mgat5	0	0	100	0
GalT	0	0	0	100

## Data Availability

The datasets generated for this study are available on request to the corresponding author.

## Author Contributions

All authors designed the study, planned the experiments, and wrote the manuscript. PF performed the experiments.

## Conflict of Interest Statement

The authors declare that the research was conducted in the absence of any commercial or financial relationships that could be construed as a potential conflict of interest.
